# The Macroscopic and Radiographic Skull and Dental Pathology of the Tasmanian Devil (*Sarcophilus harrisii*)

**DOI:** 10.3389/fvets.2021.693578

**Published:** 2021-06-10

**Authors:** Shanna Landy, Santiago Peralta, Larry Vogelnest, Nadine Fiani

**Affiliations:** ^1^Department of Clinical Sciences, College of Veterinary Medicine, Cornell University, Ithaca, NY, United States; ^2^Taronga Conservation Society Australia, Mosman, NSW, Australia

**Keywords:** Tasmanian devil, *Sarcophilus harrisii*, Dasyuridae, polyprotodont, dental anatomy, oral anatomy, dental radiography, pathology

## Abstract

While the gross skull and dental morphology, masticatory biomechanics, dental eruption patterns, and radiographic dental anatomy has been described in the Tasmanian devil (*Sarcophilus harrisii*), to date no studies have comprehensively examined the prevalence and appearance of pathologic processes affecting their skulls and dentition. As such, the aim of this study was to describe macroscopic and radiographic anatomy and identify the prevalence of anatomic variations and pathological processes in Tasmanian devil dentition and skulls. To do so, anatomical and pathological findings were documented in Tasmanian devil skulls using photography and dental radiography. Assessment of skull trauma, anatomical and developmental abnormalities, periodontitis, endodontic disease, and tooth resorption was performed. A total of 28 Tasmanian devil skulls containing 1,028 teeth were examined. Evidence of postmortem trauma was common. The most common positional abnormality was palatal or buccal rotation of the premolar teeth. While the alveolar bone margin was commonly positioned apically to the cementoenamel junction (98.2%), only 14.2% demonstrated evidence of periodontitis. Tooth fractures were common, affecting 27 skulls, however radiographic signs of endodontic disease were only noted in 4.5% of affected teeth, as was non-inflammatory root resorption (2.0%). A wider root canal width, which was used as a criterion for age determination, was associated with smaller skull dimensions, incompletely erupted teeth, and subjectively less fusion of the mandibular symphysis. Through an improved understanding of what constitutes normal anatomy and the appearance and frequency of pathologic processes that affect the skulls and teeth, this knowledge can help develop a foundation for understanding the oral health and management of live animals for this endangered species.

## Introduction

The Tasmanian devil (*Sarcophilus harrisii*) is the largest extant carnivorous marsupial in the world (1–5). While its range historically extended throughout continental Australia, it is now only found in the wild inhabiting the island state of Tasmania, located south of mainland Australia across the Bass Strait (1, 3, 4). Weighing ~8–10 kilograms for males and 6–7 kilograms for females, the Tasmanian devil is a pounce-pursuit predator, forager, and facultative scavenger (1, 5, 6). While younger devils favor a diet of smaller mammals, birds, amphibians, reptiles, and insects, as individuals age their diet shifts to primarily macropods such as Red-necked wallabies (*Notamacropus rufogriseus*) and Rufous-bellied pademelons (*Thylogale billardierii*), both as carrion and live prey (1, 2, 4, 5).

**Figure 1:**
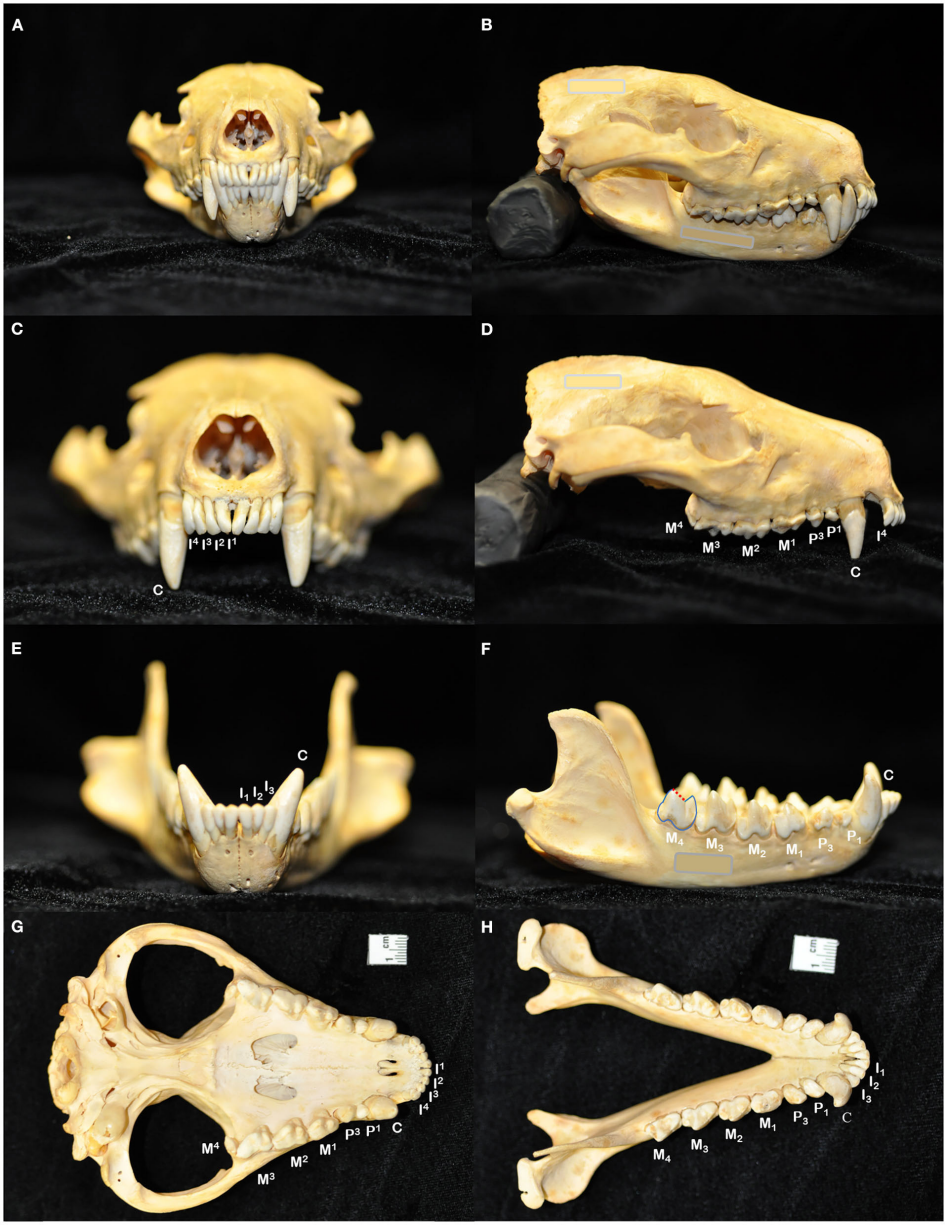
The skull of the Tasmanian devil (*Sarcophilus harrisii*) demonstrates adaptations to its carnivorous diet, including crushing the bones of its prey: a prominent midsagittal crest, broad zygomatic arches, and relatively short rostrum to exert powerful bite forces **(A,B)**. The dental formula for the Tasmanian devil is I 4/3, C 1/1, P 2/2, M 4/4, totaling 42 teeth in an adult individual **(C–H)**. In noting each tooth, I, incisor tooth; C, canine tooth; P, premolar tooth; M, molar tooth. Superscripts denote maxillary teeth while subscripts are used to number mandibular teeth. While the maxillary molar teeth bear a crest and occlusal basin design that is conducive to crushing **(D,G)**, the crowns of the mandibular molar teeth each have a paracristid crest (red dotted line) between the paraconid and metaconid cusps, creating a sharp slicing blade and notch similar in form and function to the carnassial edge of placental carnivores **(F,H)**.

The pronounced midsagittal crest, broad and widely spaced zygomatic arches, and overall thicker maxillofacial bones of the Tasmanian devil relative to phylogenetically similar species accommodate powerful masticatory musculature that, together with the relatively short rostrum, generates exceptionally strong bite forces capable of crushing the bones of their prey (4, 7–9) (Figures 1A,B). Additional unique orofacial properties of the Tasmanian devil include a postorbital process of the frontoparietal bone that partially partitions the globe of the eye from the zygoma, and the absence of an articular disc within the temporomandibular joint, which instead contains a shock-absorbing thick fibrous tissue covering the surfaces of the articular condyle and mandibular fossa (4, 10).

**Figure 2:**
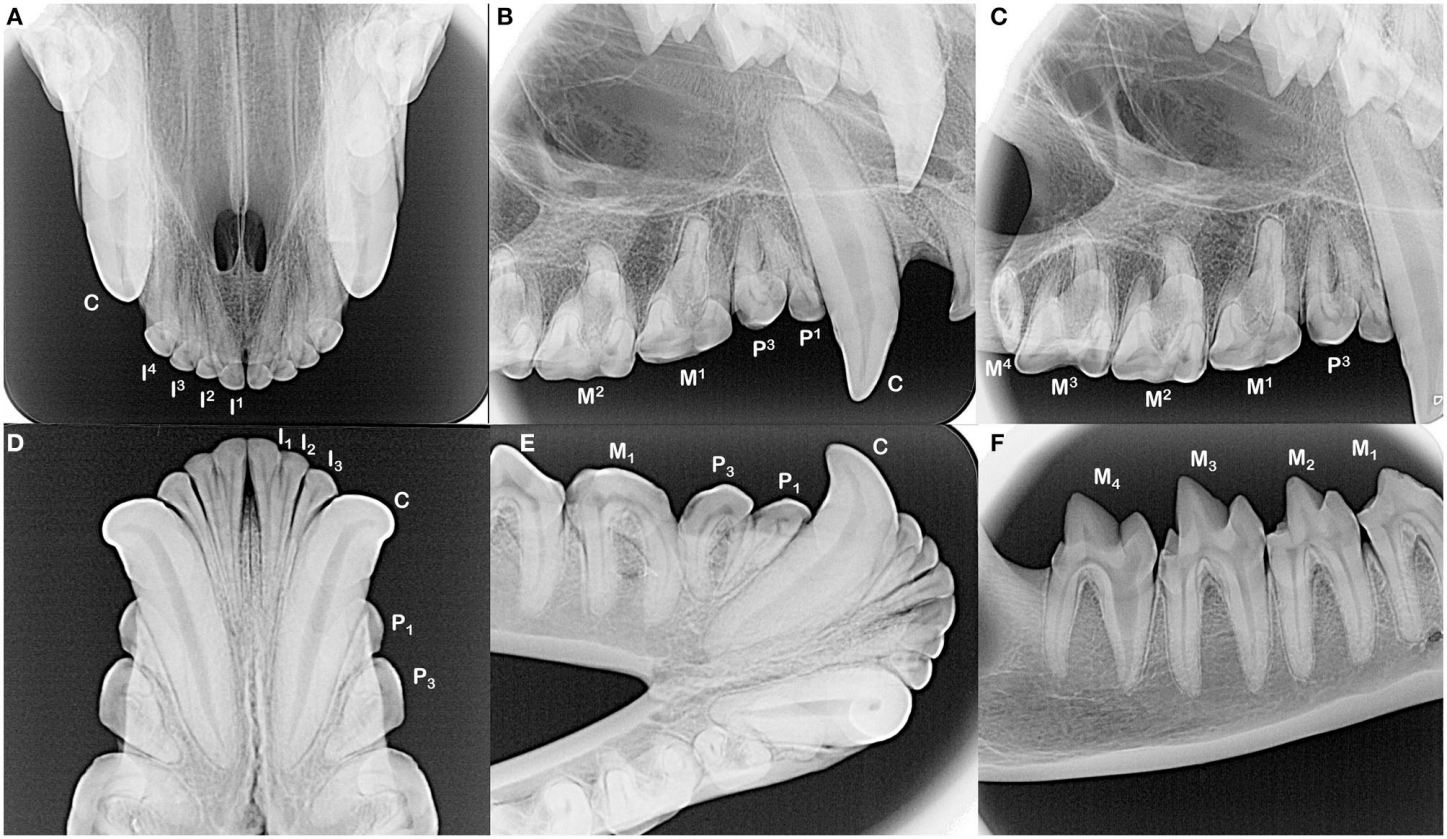
The incisors and canine teeth are single rooted **(A,B,D,E)**. Each of the maxillary and mandibular premolar teeth in each quadrant have two roots, although distinction of these roots can be complicated by their convergence and/or rotation of the teeth **(B,E)**. All maxillary molar teeth have three roots, which can be difficult to identify in the fourth molar tooth as these roots converge and may fuse (**C**, note dilaceration of the palatal root on the second molar tooth). All mandibular molar teeth have two roots **(E,F)**. The root canal of the mesial root of the fourth mandibular molar tooth is notably wider compared to the distal root.

The dentition of the Tasmanian devil is also adapted to a hypercarnivorous diet (11, 12). The Tasmanian devil has a total of 42 teeth, represented by the dental formula I 4/3, C 1/1, P 2/2, M 4/4 (4, 13–15) (Figures 1C–H). They are all bunodont, with a short crown and well-developed root structure, and the crowns of nearly all teeth are covered with enamel to the level of the gingival margin, except for the incisor and canine teeth where enamel only covers the coronal two thirds of the crown (4, 16). The incisor teeth are polyprotodont, which describes marsupials possessing four or more incisor teeth in each maxilla and more than two incisor teeth in each mandible and is one of the distinguishing apomorphies of the Dasyuridae. The maxillary incisor teeth are oriented transversely, permitting relatively rostral positioning of the strong, cylindrically based, grossly enlarged canine teeth to facilitate grasping of large prey (4, 15, 17). There are only two maxillary and mandibular premolar teeth in Tasmanian devils, reduced from three in the ontogenetic ancestral marsupial, which have been identified as the first and third premolar teeth despite this labeling being inconsistent with standard terminal reduction theory (14–16). These teeth are small, and the cusps of the maxillary and mandibular counterparts do not normally engage in occlusal contact when the jaw is closed. The maxillary molar teeth are bunodont, with paracone, metacone, and protocone cusps enclosing an occlusal basin that makes these teeth ideal for crushing (14, 16) (Figures 1D,G). In contrast, each mandibular molar tooth has a paracristid crest between the paraconid and metaconid cusps, creating a sharp slicing blade and notch similar in form and function to the carnassial edge of placental carnivores (4, 14, 16) (Figures 1F,H). The incisor and canine teeth each have a single root (Figures 2A,B,D,E). The premolar teeth are two-rooted, although rotation and convergence of the roots of the first premolar tooth can complicate distinction between them and their independent radiographic interpretation (16) (Figures 2B,E). The maxillary molar teeth all have three roots, although the fourth molar tooth is smaller than its counterparts and has convergent roots, at times giving it the appearance of having only a single root (16) (Figure 2C). The molar teeth on the mandibular arch all have two distinct roots (16) (Figures 2E,F).

Numerical and positional dental anomalies have been identified in Tasmanian devils, including incisor tooth crowding, lateral rotation of the third premolar tooth, and crown abnormality of a second premolar tooth (13, 18–20). In a separate specimen, a left maxillary third premolar tooth, paired supernumerary mandibular premolar teeth, an unpaired right maxillary molar tooth, a single supernumerary maxillary molar and paired supernumerary mandibular molar teeth, crown division with possible extension into the root of a left maxillary fourth molar tooth, and bilaterally very small maxillary fourth molar teeth were identified (21). However, these previous documentations were limited to evaluation of crown presence and position in intact skulls and did not include description of trauma to or structural abnormalities of the crowns themselves. While the normal radiographic dental anatomy in Tasmanian devils has also been described (16), to the authors' knowledge there is no published data regarding pathological radiographic findings in Tasmanian devils, such as those of developmental, periodontal, or endodontic tooth disease or other abnormalities of the alveolar bone. As such, the aim of the present study was to identify the prevalence of and describe macroscopic and radiographic anatomy, anatomic variations, and pathological processes involving the skulls and dentition of a museum collection of Tasmanian devil skulls.

## Materials and Methods

A total of 30 Tasmanian devil skulls were examined from the collection of the Australian Museum using photography and veterinary dental radiographic equipment at the Taronga Zoo, both located in Sydney, Australia. Of these specimens, 28 were intact skulls containing entire maxillae and mandibles and were thus included in this analysis. Specimens were cataloged by the museum collection with individual identification numbers, as well as the date of recovery and sex of the animal, if known. None of the specimens had the precise age of the animal recorded.

The maxillae and mandibles were visually inspected and photographed in a standardized manner, both articulated and separately, across eleven images capturing the frontal, lateral, and occlusal planes of view, specifically from the ventral aspect of the maxilla and dorsal aspect of the mandibles. When notable pathology was identified, such as overt crown fracture, focally severe recession of the alveolar bone, or marked dental malocclusion, additional targeted photographs were obtained. All photographs were taken using a Nikon D90 digital-SLR camera with a Nikon 60 mm 1:2.8D AF Micro-Nikkor lens (Nikon, Tokyo, Japan) at a horizontal and vertical image resolution of 300 dpi. Images were analyzed and measurements were obtained using an open-source image processing software [Fiji Is Just ImageJ, (21)].

**Figure 3:**
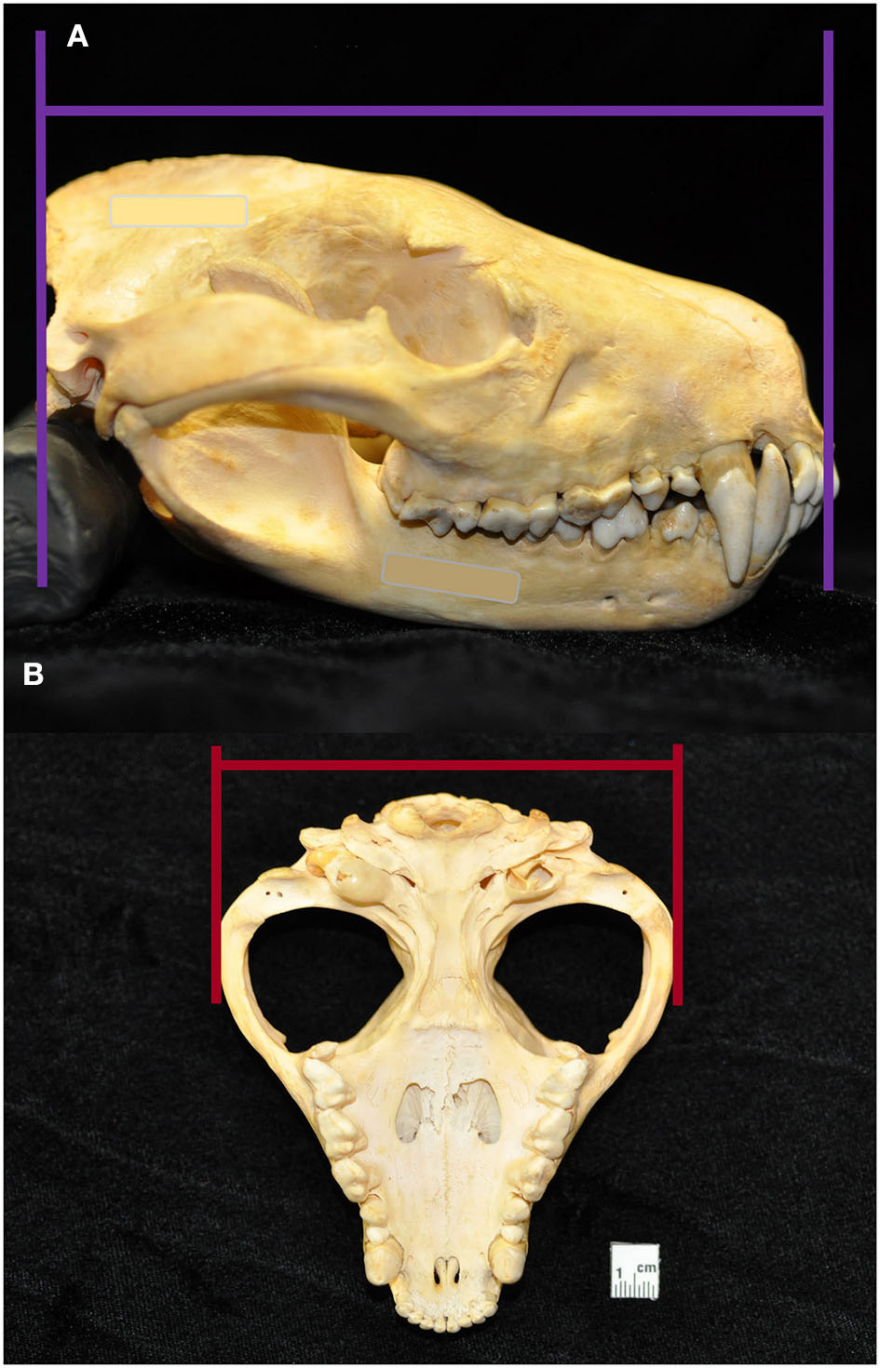
Skull length was measured in the lateral plane as a straight line parallel to the hard palate, between the rostral end of the interincisive suture to the central point of the occipital protuberance **(A)**. Skull width was measured in the ventral plane as a straight line parallel to the hard palate, between the widest points of the lateralmost surface of the left and right zygomatic **(B)**. From these measurements, skull index was calculated as the skull width multiplied by 100 and divided by the skull length.

Macroscopic measurements and assessment of the teeth and associated hard tissues of the jaws were performed using predetermined objective criteria, summarized in Table 1, using modified assessment criteria for dry skull dental pathology (22). Skull length and width measurements were adapted from previous methods on measuring the condylobasal length and zygomatic arch width of Australian dasyurid carnivore skulls (23). Skull length was instead measured in the lateral plane as a straight line parallel to the hard palate, between the rostral end of the interincisive suture to the central point of the occipital protuberance. Skull width was measured in the ventral plane as a straight line parallel to the hard palate, between the widest points of the lateralmost surface of the left and right zygomatic arch (Figure 3). Skull index was calculated as the skull width multiplied by 100 and divided by the skull length. The presence of skull fractures, palatal suture or mandibular symphyseal separation, presence of artificial manipulation such as drilled holes or the placement of wire around skull elements, relative size of the palatine vacuities and palatine fissures, subjective degree of loss of the nasal turbinate architecture, and any other anatomical abnormalities of the skull were described.

**Table 1:** Evaluation criteria for anatomical and pathological innate and acquired findings.

Skull	
Skull length	Measured in cm in the lateral plane as a straight line parallel to the hard palate, between the rostral end of the interincisive suture to the central point of the occipital protuberance.
Skull width	Measured in cm in the ventral plane as a straight line parallel to the hard palate, between the widest points of the lateralmost surface of the left and right zygomatic arch
Skull index	Calculated as the skull width multiplied by 100 and divided by the skull length
Maxillofacial damage	Fractures or abnormal wear of the skull or alveolar bone, maxillary suture or mandibular symphyseal separation, subjective degree of loss of nasal turbinate architecture (mild, moderate, or severe), presence of defects suspected to be secondary to historical preparation for display
Miscellaneous maxillofacial findings	Symmetry of the palatine vacuities and palatine fissures, deviation of the nasal septum, degree of mandibular symphyseal fusion demonstrated radiographically (unfused, partial, or complete)
Dental – anatomical and developmental	
Unable to evaluate	Tooth absence accompanied by fracture and loss of the alveolar bone
Congenital absence	Tooth absence with smooth, morphologically normal bone present at the site of the alveolus with no overt evidence of acquired tooth loss
Acquired absence – antemortem	Tooth absence with pathologic changes to the alveolar bone such as rounding of the alveolar margin, loss of depth of the alveolus, periosteal reaction, or increased vascular foramina
Acquired absence – postmortem	Tooth absence with a morphologically normal sharply marginated alveolus demonstrating no signs of pathologic changes to the bone
Dental malocclusion	Tooth position is rotated compared to its contralateral counterpart or deviated from the main axis of the crowns of adjacent teeth.
Malformation	Presence of an abnormally shaped or abnormally mineralized crown
Supernumerary tooth	Presence of an extra tooth
Persistent deciduous tooth	Presence of a deciduous tooth when it should have exfoliated, in this case in the presence of a permanent counterpart
Infraerupted tooth	Location of the crown of the tooth with the cementoenamel junction partially below the margin of the surrounding alveolar bone
Unerupted tooth	Location of the crown of the tooth with the cementoenamel junction completely below the margin of the surrounding alveolar bone
Supernumerary root	Presence of an extra root
Root convergence	Abnormal angulation or positioning of the roots toward one another
Root divergence	Abnormal angulation or positioning of the roots away from one another
Root dilaceration	Abnormal bending or crookedness of the root
Root canal width	Measured percentage of the width of the whole root of the maxillary canine teeth halfway between the cementoenamel junction and apex of the tooth, averaged between the left and right maxillary canine teeth when possible
Dental – periodontal disease	
Alveolar bone recession	Location of the margin of the alveolar bone apical to the level of the cementoenamel junction
Asymmetrical alveolar bone recession	Greater recession of alveolar bone from the cementoenamel junction of one tooth relative to its contralateral partner
Furcation involvement/exposure	Partial/full-thickness loss of alveolar bone at the furcation of a tooth
Alveolar bone expansion	Thickening of the alveolar bone at the buccal and labial aspects of a tooth
Alveolar bone fracture	Fracture affecting the bone forming the alveolus of a tooth
Luxation	Displacement of a tooth from or within its alveolus
Dental – endodontic disease	
Attrition/abrasion	Tooth wear caused by contact of a tooth with another tooth/with a non-dental object
Enamel fracture	Fracture with loss of crown substance confined to the enamel
Uncomplicated crown fracture	Fracture of the crown that does not involve the pulp
Complicated crown fracture	Fracture of the crown that exposes the pulp
Uncomplicated crown-root fracture	Fracture of the crown and root that does not expose the pulp
Complicated crown-root fracture	Fracture of the crown and root that exposes the pulp
Root fracture	Fracture involving the root, may or may not be associated with a clinically absent crown
Linear fracture	Fracture line extending from the surface of the crown into the tooth, not associated with a loss of crown integrity
Macroscopic periapical lesions	Macroscopic compromise of the alveolar bone overlying the periapical region, such as focal periosteal reaction or sinus tract formation
Failure of the root canal to narrow	Subjectively relatively wider root canal width compared to contralateral counterpart or relative to similar adjacent teeth
Periapical lucency	Halo-like loss of alveolar bone density centered around the apex
Inflammatory root resorption	Loss of dental tissues appearing as blunting or shortening of the root apex or elliptical enlargement of the root canal in the presence of other signs of endodontic disease
Dental – Non-inflammatory resorption	
External surface	Shallow resorption lacunae affect the cementum and dentin, located along the lateral margins of the root and not affecting the periodontal ligament space or lamina dura
External replacement	Gradual effacement of the periodontal ligament space with progressive replacement of the root tissues by alveolar bone
External cervical root surface	Invasive resorption beginning at the cervical area of the tooth and progressing coronally and apically
Internal surface	An elliptical enlargement of the apical third of the root canal
Internal replacement	Irregular enlargement of a tunnel-like appearance adjacent to the root canal

Each tooth's presence, or suspected reason for its absence, was noted as well as any overt malocclusive positioning. While occlusal assessment has yet to be standardized in Tasmanian devils as it has been in small animal patients, an attempt was made to identify any maloccluded teeth that were abnormally rotated or deviated compared to its contralateral counterpart and/or relative to the crowns of adjacent teeth or regions of abnormal crowding (24). The presence and position of a supernumerary tooth or persistent deciduous tooth adjacent to a permanent counterpart was noted. Because all specimens, including suspect juveniles, demonstrated an alveolar bone margin that did not reach the cementoenamel junction of nearly all teeth examined, it was separately noted whether individual teeth had comparatively greater alveolar bone recession relative to contralateral counterparts. Attritional or abrasive wear of the teeth resulting in blunting of the tips of the cusps or the paracristid crest, was also documented. Fractures of the teeth were categorized according to the depth of the dental hard tissues affected as well as its involvement of the crown, root(s), and/or both according to a well-established classification scheme routinely used in small animal dental practice (25). Finally, any evidence of periapical pathology or miscellaneous maxillofacial findings were noted.

**Figure 4:**
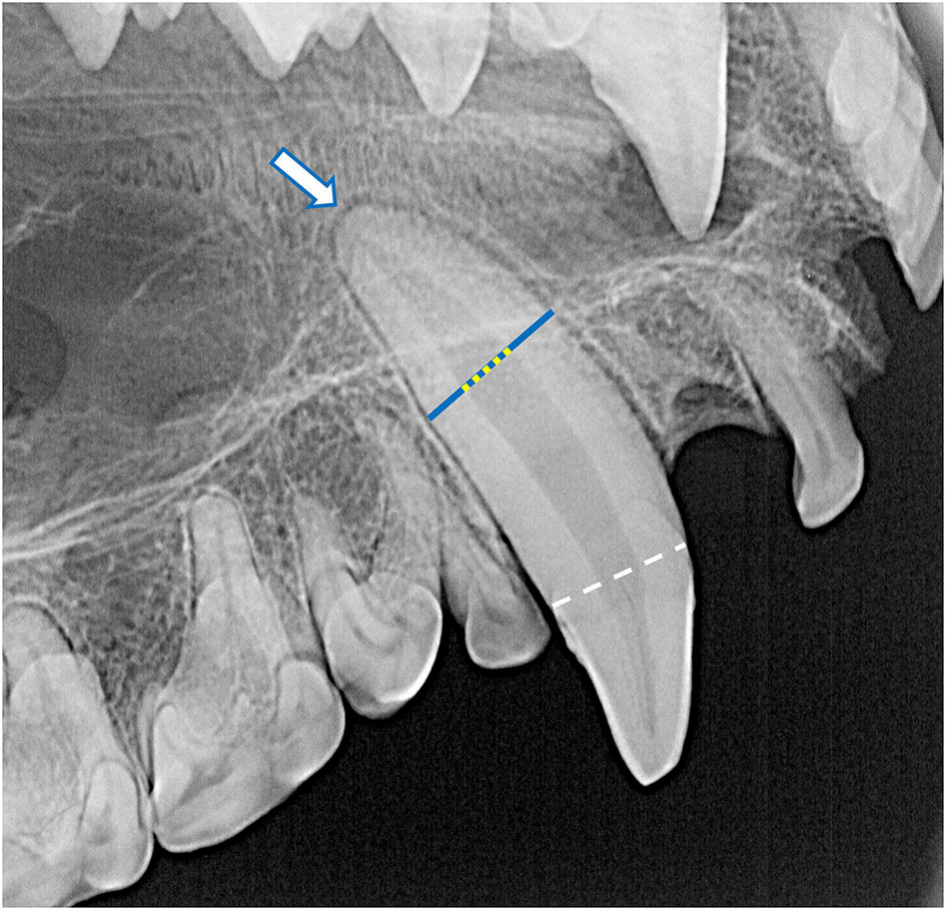
The root canal width (yellow dotted line) was calculated as the percentage of the width of the whole root (blue line) of the maxillary canine teeth halfway between the cementoenamel junction (white dashed line) and apex of the tooth (white arrow). For each specimen, mean root canal width was measured from both maxillary canines. If a maxillary canine tooth was missing, demonstrated signs of endodontic disease, or was fractured below the level of the cementoenamel junction, the other canine tooth was solely measured. No skulls had bilateral compromise of maxillary canine teeth that would have precluded this assessment. In this case, the whole root width was measured as 680 pixels and the root canal width was measured as 236 pixels, resulting in a root canal width of 34.7%.

**Figure 5:**
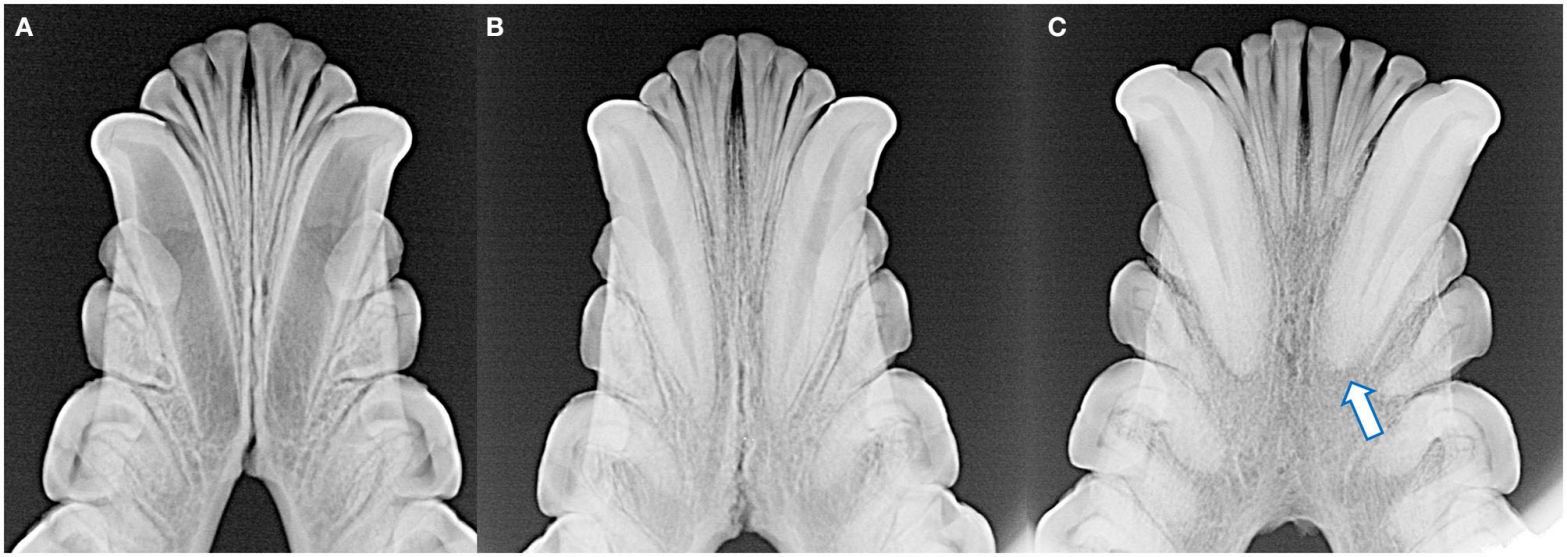
The degree of mandibular fusion was subjectively categorized into three classifications: unfused **(A)**, partial fusion **(B)**, and complete fusion **(C)** according to the thickness and length of the mandibular symphysis visible radiographically. Note the open apices of the mandibular canine teeth and relatively wide root canals of the skull with the unfused symphysis **(A)**, as well as external replacement resorption of the left mandibular canine tooth (arrow) of the skull with the completely fused symphysis **(C)**.

Dental radiographs were obtained using a portable dental radiography unit (Portable X-ray II, 60 kV/2 mA, Genoray Co., Ltd., Gyeonggi-do, Republic of Korea) and images were processed using a computed radiography plate scanner (CR 7 Vet Image Plate X-ray Scanner, iM3®, Vancouver, WA, USA). Maxillary and mandibular occlusal, left and right lateral canine-to-premolar, and caudal premolar and molar tooth views were obtained for each specimen. A parallel technique was used for radiographing the teeth of the caudal mandibles, and the intraoral bisecting angle technique was used to obtain all other views (16). The presence of the tooth and any positional abnormalities including unerupted teeth were noted. The tooth roots were evaluated for total number, concrescence, convergence, fusion, convergence, divergence, and/or dilaceration. The periodontal status of each tooth was initially assessed using a classification scheme adapted for application in dry skulls, which classifies the degree of periodontitis into three different stages depending on the depth and pattern of alveolar bone loss among other factors (26–28). However, because the presence of minimal interproximal bone was very limited due to close spacing of the teeth, as well as the near ubiquity of the alveolar bone margin not reaching the cementoenamel junction, use of this classification scheme was discontinued. Instead, radiographic assessment of alveolar bone height was limited to complementing visual assessment of relative asymmetrical alveolar bone recession and furcation involvement or exposure. Radiographic signs of endodontic disease, such as loss of crown integrity, failure of the pulp cavity to narrow compared to its contralateral counterpart and/or adjacent teeth, the presence of a periapical lucency, and suspect inflammatory root resorption were recorded (28). To classify the age of the specimen at the time of collection, the width of the root canal was standardized as a percentage of the width of the whole root of the maxillary canine teeth halfway between the cementoenamel junction and apex of the tooth. This was measured and averaged between the left and right maxillary canine teeth in each specimen (Figure 4). If a maxillary canine tooth was missing, demonstrated signs of endodontic disease, or was fractured below the level of the cementoenamel junction, the other canine tooth was solely measured. No skulls had bilateral compromise of maxillary canines that would have precluded this assessment. Tooth resorption identified independently of suspect endodontic disease was noted and classified according to previously established criteria for companion canine patients (28, 29). Evidence of alveolar bone fracture or other dentoalveolar trauma was also recorded. Finally, the subjective degree of fusion of the mandibular symphysis (none, partial, or complete) was noted (Figure 5).

Data analysis was performed using commercially available spreadsheet (Excel 365, Microsoft Co., Redmond, WA, USA) and statistical analysis software (GraphPad Prism 9, GraphPad Software, San Diego, CA, USA). The significance level (*p*-value) for all analyses was set at α = 0.05. Categorical data was described as the frequency of occurrence. Numerical data was summarized with mean values listed ± one standard deviation from the mean. Fischer's exact test was used to investigate the relationship between pairs of categorical variables. Pearson's correlation coefficient was used to investigate correlations between numerical variables. Student's *t*-test was used to assess for differences in skull length, width, and index between known and suspected female specimens compared to the overall population, as well as to compare differences in root canal width in skulls with and without incompletely erupted teeth. One-way ANOVA with a *post-hoc* Tukey test was used to investigate differences in root canal width in skulls with varying degrees of mandibular symphyseal fusion.

## Results

Twenty-eight *Tasmanian devil* skulls were examined, with a total of 1,028 teeth present in whole or in part available for examination. Three of the skulls were labeled as female in the accompanying museum records, with an additional three described as potential females; the remaining 22 did not have a known or suspected sex identified. Twenty-two of the specimens were recovered in the year 1866, with an additional five having been recovered after that date and one with an unknown recovery date. The average skull length and width were 12.3 (±1.0) and 9.7 (±0.9) respectively, with a mean skull index of 79.2 (±4.9). No significant difference was identified when the skull measurements were assessed specifically among the known and suspected females compared to the rest of the study population (skull length 11.9 ± 0.5 *p* = 0.302, skull width 9.6 ± 0.4 *p* = 0.701, skull index 80.7 ± 6.5 *p* = 0.371).

**Figure 6:**
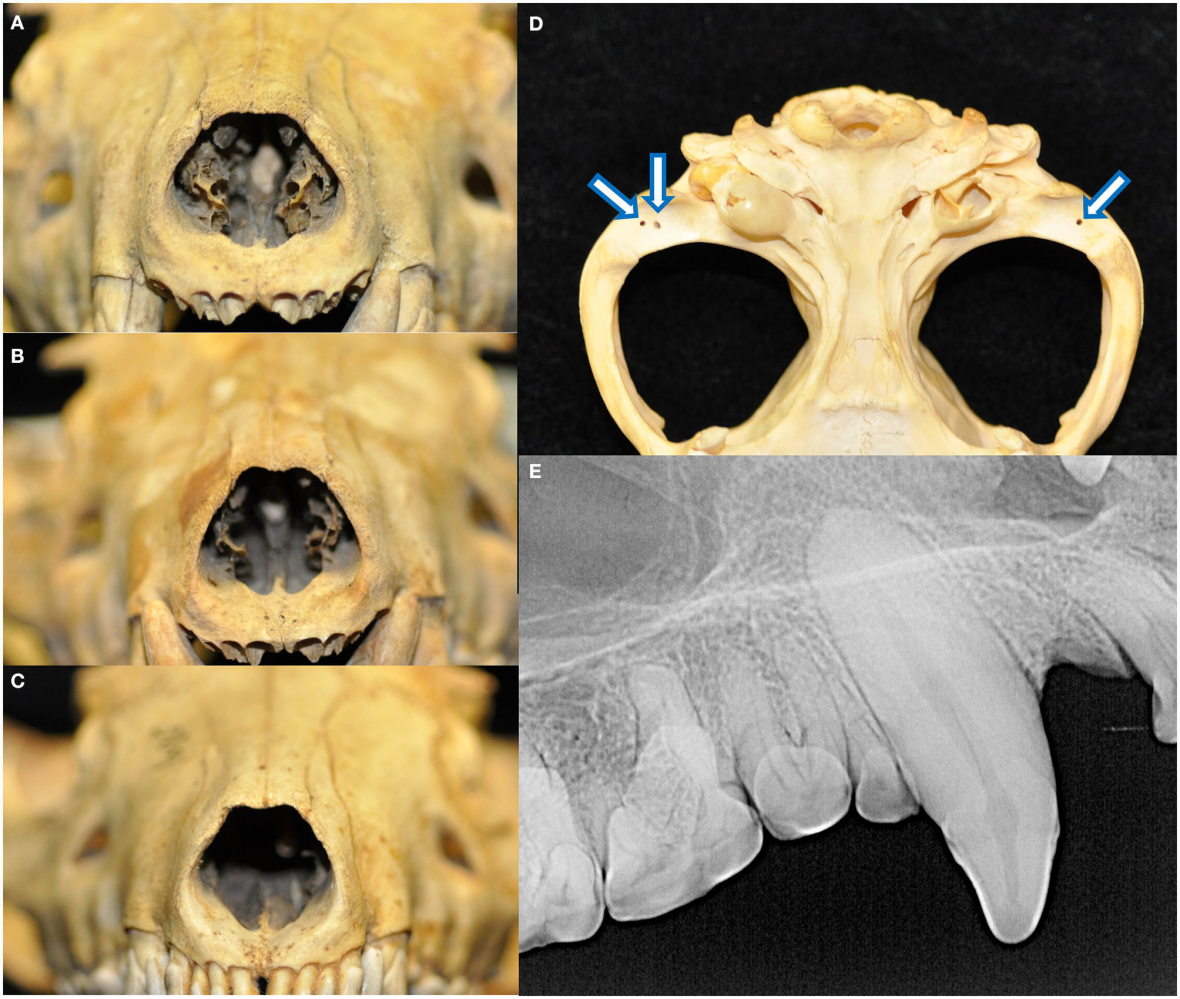
All skulls evaluated demonstrated changes attributed to postmortem handling. Loss of nasal turbinate structure was noted in all specimens, including subjectively mild **(A)**, moderate **(B)**, and severe **(C)** degrees of affectedness. Approximately 1-by-1 mm circular shallow defects were identified in the mandibular fossae of the temporal bones and condylar processes of the mandibles **(D)**. These were perhaps historically used as seating points for peg or wires for mounting and display apparatuses. Finally, fracture lines extending parallel to the long axis of the tooth with no associated radiographic evidence of endodontic disease was noted in 46 teeth **(E)**.

The palatine fissures were symmetrical or nearly symmetrical in eighteen specimens, while the right was larger in five specimens and the left was larger in an additional five. The palatine vacuities were symmetrical or nearly symmetrical in eleven specimens, with eight having a larger right vacuity, eight having a larger left vacuity, and one specimen demonstrating vacuities of an overall subjectively similar size but differing shape. All skulls exhibited loss of nasal turbinate structure, with five subjectively categorized as mild, seven moderate, and the remaining sixteen as severe (Figures 6A–C). Two skulls demonstrated left lateral deviation of the nasal septum but no deviation or deformation of the bones of the overlying nasal bridge. In one skull, the right condylar process of the mandible appeared to be excessively worn, with a subjectively shallower right mandibular fossa and blunted retroarticular process.

Eighteen specimens had fractures of the skull, all of which had sharply demarcated separation lines and no evidence of callus or remodeling. Fourteen of these skulls bore fractures to the ventral aspects of the tympanic bullae, either unilaterally (three of the right, two of the left) or bilaterally (nine). Other fractures and separations were observed as follows: the alveolar juga of the missing left maxillary canine tooth; alveolar bone fractures distal to the left mandibular third premolar and right mandibular first premolar teeth; right mandibular fossa and zygomatic process of the temporal bone, as well as the alveolar jugae of the left maxillary third premolar to first molar teeth; left retroarticular process; the retroarticular processes bilaterally; buccal alveolar bone fracture of the right maxillary first and second incisor teeth; the left retroarticular process and right coronoid process; comminuted right frontal bone fracture affecting the calvarium and medial orbit, as well as mandibular symphyseal separation held together with a bridging wire fastened to the ventral cortices; and maxillary suture separation along with a fractured dorsal nasal bridge. Approximately one-by-one-millimeter shallow circular defects that may have been historically used as seating points for mounting and display apparatuses were found bilaterally in the mandibular fossae of the temporal bones of 26 specimens and in the condylar processes of the mandibles in 22 specimens (Figure 6D). Linear fractures, identified as the presence of a fracture line along the long axis of the tooth not associated with any loss of tooth material or radiographic evidence of endodontic disease, were found in 46 teeth (Figure 6E). Because these defects were strongly suspected to be strictly artifactual in nature and were presumed to have occurred during postmortem handling, they were excluded from further analysis on periodontal and endodontic disease.

Radiographically, the mandibular symphysis was found to undergo variable degrees of fusion across different specimens. This was subjectively categorized as complete fusion (*n* = 10), partial fusion (*n* = 13), and no evidence of fusion (*n* = 4). One skull had undergone mandibular symphyseal separation and the mandibles were artificially joined with wire, and as such the degree of previous mandibular symphyseal fusion could not be determined.

A total of 148 teeth were determined to be clinically absent relative to the standard dental formula for the Tasmanian devil. The most common absent teeth were the maxillary and mandibular incisor teeth, accounting for 111 (75%) of missing teeth. Most teeth (*n* = 138, 93.2%) were associated with a clearly defined and sharply marginated alveolus and were suspected to be lost postmortem. In six cases of missing teeth, there were visual or radiographic changes to the alveolar bone that could explain potential antemortem loss of attachment and subsequent loss either during life or postmortem handling. The only teeth identified as being absent with no radiographic appearance of a vacant alveolus were maxillary fourth molar teeth that were missing bilaterally in two specimens that also demonstrated features suggestive of a young age at the time of death, such as diffusely subjectively wide pulp cavities, incompletely erupted teeth, and unfused mandibular symphyses.

Of the 1,028 teeth remaining in this collection, 191 teeth demonstrated positional, anatomical, or other suspect developmental anomalies. The most common positional abnormality according to the predetermined criteria was rotation of the mesial aspect of the maxillary premolar teeth toward the palate, seen in 72 (68%) of these teeth, and rotation of the mesial aspect of the mandibular premolar teeth toward the buccal surfaces, seen in all 106 mandibular premolar teeth available for evaluation. Other noted dental malocclusions included buccal rotation of right maxillary first (*n* = 1) and second (2) incisor teeth; mesioversion of a right maxillary first incisor tooth (*n* = 1), left maxillary first (*n* = 1) and second (1) incisor teeth; buccoversion of a right maxillary first incisor tooth (*n* = 1) and right mandibular first incisor tooth (*n* = 1); and linguoversion of two mandibular second incisor teeth, one of which also had a morphologically abnormal crown. Maloccluded teeth were found to be associated with relative asymmetrical alveolar bone loss at a frequency of 19.4%, compared to 13.2% of normally positioned teeth (*p* = 0.043). Furcation involvement or exposure was identified in 25.1% of maloccluded multirooted teeth, but only 5.6% of normally positioned multirooted teeth (*p* < 0.0001). No statistically significant difference was found between maloccluded and normally positioned teeth with regards to signs of endodontic disease (*p* = 0.153).

**Figure 7:**
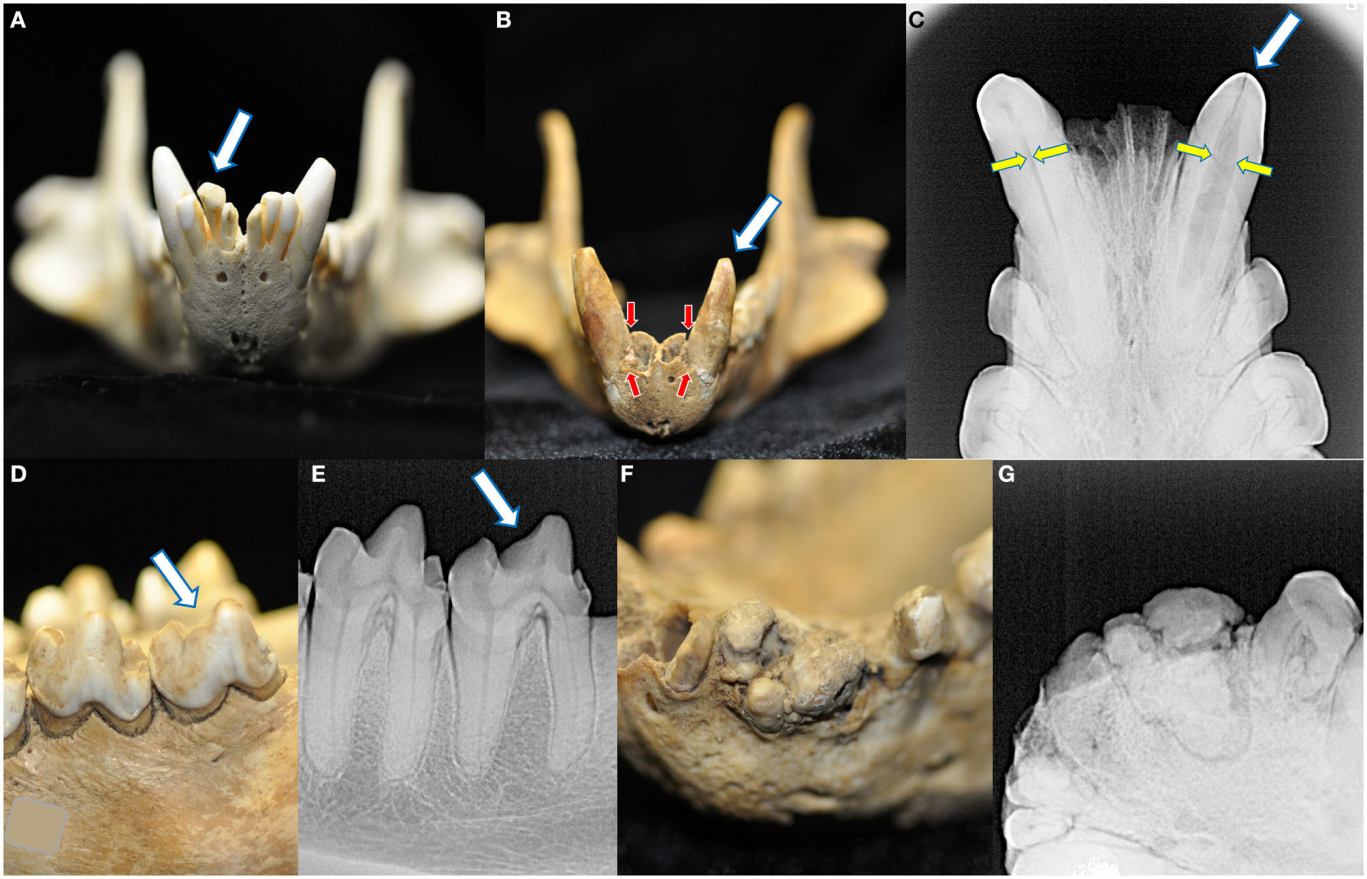
Four macroscopic and radiographic structural abnormalities of the crowns were identified in this collection. A linguoverted right mandibular second incisor tooth had an enlarged and bulbous-shaped crown **(A)**. One skull had two crown abnormalities. The first was a left mandibular canine tooth that was smaller and more conical than its right mandibular counterpart **(B)**, with severe alveolar bone loss (red arrows, compared to the right mandibular canine tooth) and failure of the root canal to narrow (yellow arrows) **(C)**. Please note this tooth also bears a linear fracture. The same specimen also had a left mandibular fourth molar tooth with a blunted paracristid crest **(D)** but no radiographic evidence of endodontic disease **(E)**. Finally, one specimen had a grossly abnormal arrangement of the left mandibular second incisor through the first premolar tooth **(F)**, demonstrating irregular mineralization, crown and root fusion, and failure of normal root and periodontal development **(G)**.

Across all specimens, four cases of abnormalities in crown structure were identified (Figure 7). No retained, persistent deciduous, or supernumerary teeth were identified in this collection. No enamel hypoplasia was observed.

A noteworthy anatomical radiographic finding in these Tasmanian devil specimens is that the mesial root of the mandibular fourth molar tooth is relatively thicker and has a correspondingly wider root canal compared to the distal root, demonstrated in all fourth mandibular molar teeth of the examined specimens. The total number of roots and potential degree of fusion was difficult to determine in the maxillary fourth molar teeth due to the relatively small size of the tooth and convergence of its roots, as well as the palatoverted maxillary premolars and buccoverted mandibular premolars. However, no overt supernumerary or completely converged roots were noted. A total of fourteen dilacerated roots were identified: the mesial root of a right maxillary third premolar tooth, and the palatal root of the maxillary second (*n* = 11) and third (*n* = 2) molar teeth.

Of the 993 teeth that had a supragingival component, meaning exclusion of those that were clinically absent or had root fractures with missing coronal segments, 975 (98.2%) were surrounded by alveolar bone that did not reach coronally to the cementoenamel junction. Nine teeth were incompletely erupted with crown enamel located beneath the alveolar bone margin on radiographs: a right and left maxillary third molar tooth and left and right mandibular fourth molar teeth in one skull, and a right and left maxillary first incisor tooth, left mandibular second molar tooth, and left and right mandibular first molar teeth in a second skull. These two skulls bore features indicative of a young age at death, namely that these specimens had the smallest skull lengths, smallest skull widths, and largest maxillary canine root canal widths out of the collection. The maxillary third molar teeth in the first specimen demonstrated radiographic evidence of endodontic disease that may have terminated appropriate eruption: failure of the root canals to narrow relative to adjacent similarly sized molars and periapical lucencies. The remaining nine teeth were all maxillary fourth molars in which the alveolar bone was located at the level of the cementoenamel junction.

Due to the near ubiquity of having alveolar bone margins located apically to the cementoenamel junctions, relative alveolar bone loss between contralateral counterparts and alveolar margin recession resulting in furcation involvement or exposure was categorized as evidence of pathological bone loss. Asymmetrical relative alveolar bone recession was identified in 141 of 992 (14.2%) fully erupted teeth with an intact supragingival component, the majority of which were premolar (*n* = 25, 31.9%) or molar teeth (*n* = 67, 47.5%). Involvement or exposure of the furcation was seen in 54 of 204 (26.4%) of premolar teeth and 24 of 428 (5.6%) of molar teeth.

A loss of crown integrity or fracture line was observed in 693 (67.4%) teeth. Of these, 559 (80.7%) had blunt or rounded defect margins limited to the occlusal surfaces and were subjectively categorized as secondary to abrasive wear. The most frequently abraded teeth were the mandibular molar teeth, with 143 abraded teeth representing 66.2% of the total present mandibular molar teeth. This was followed by the maxillary (*n* = 33) and mandibular canine teeth (*n* = 32), representing 64.7 and 62.7% of their respective populations, then the mandibular premolar (*n* = 64, 60%), maxillary premolar (*n* = 59, 55.7%), maxillary molar (*n* = 113, 52.5%), mandibular incisor (*n* = 60, 45.5%), and maxillary incisor teeth (*n* = 52, 34.9%). No teeth were categorized as having attritional wear because no overt tooth-on-tooth malocclusive trauma or changes were identified when the mouths were closed except for the specimen with the bulbously malformed right mandibular second incisor crown that was associated with buccal rotation of the right maxillary first and second incisor teeth.

The most common fractures identified were uncomplicated crown fractures (*n* = 32), followed by root fractures (*n* = 25), complicated crown fractures (*n* = 23), complicated crown-root fractures (*n* = 5), uncomplicated crown root fractures (*n* = 3), and enamel fractures (*n* = 2). The most commonly fractured teeth were the mandibular (*n* = 33, 37.5%) and maxillary incisor teeth (*n* = 22, 25.%), followed by the maxillary canine and maxillary molar teeth (each *n* = 11, 12.5%), and maxillary premolar, mandibular canine, mandibular premolar, and mandibular molar teeth (each *n* = 6, 6.8%). One maxillary canine tooth with a complicated crown fracture and one maxillary canine tooth with an uncomplicated crown fracture also had linear fractures.

**Figure 8:**
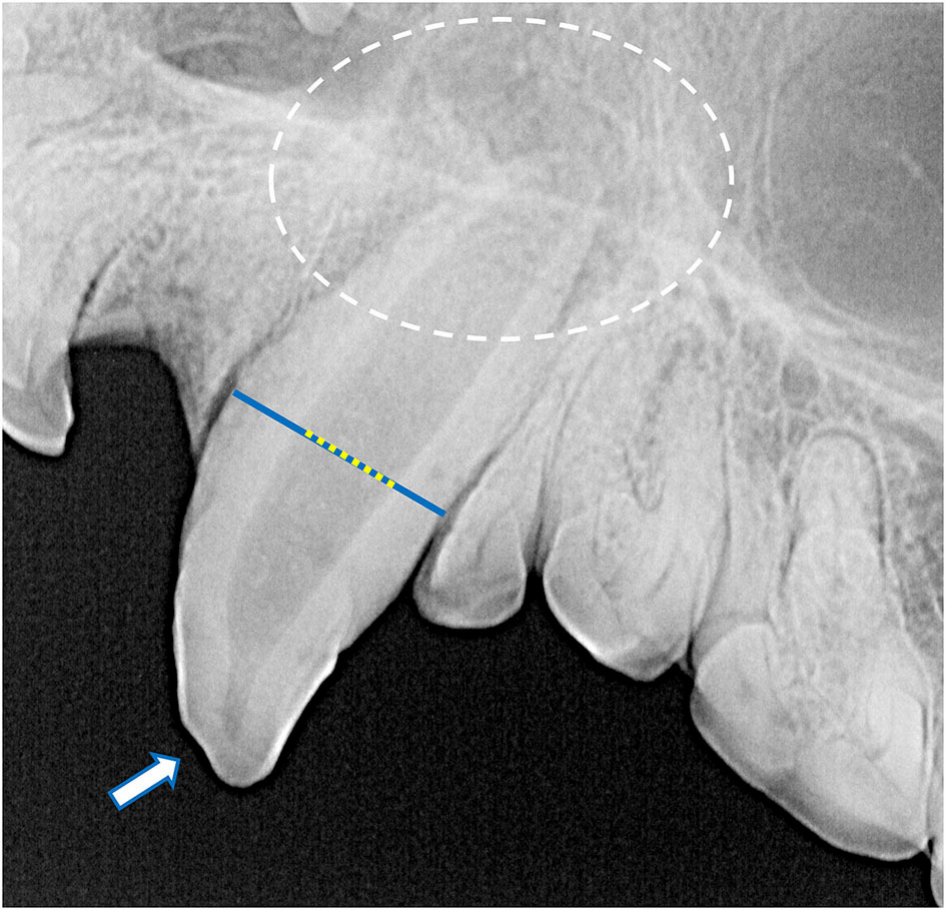
Radiographic signs of endodontic disease demonstrated in these specimens included a loss of crown integrity (white arrow), failure of the root canal to narrow (yellow dotted line overlying a blue line), periapical lucency and inflammatory root resorption (white circle).

**Figure 9:**
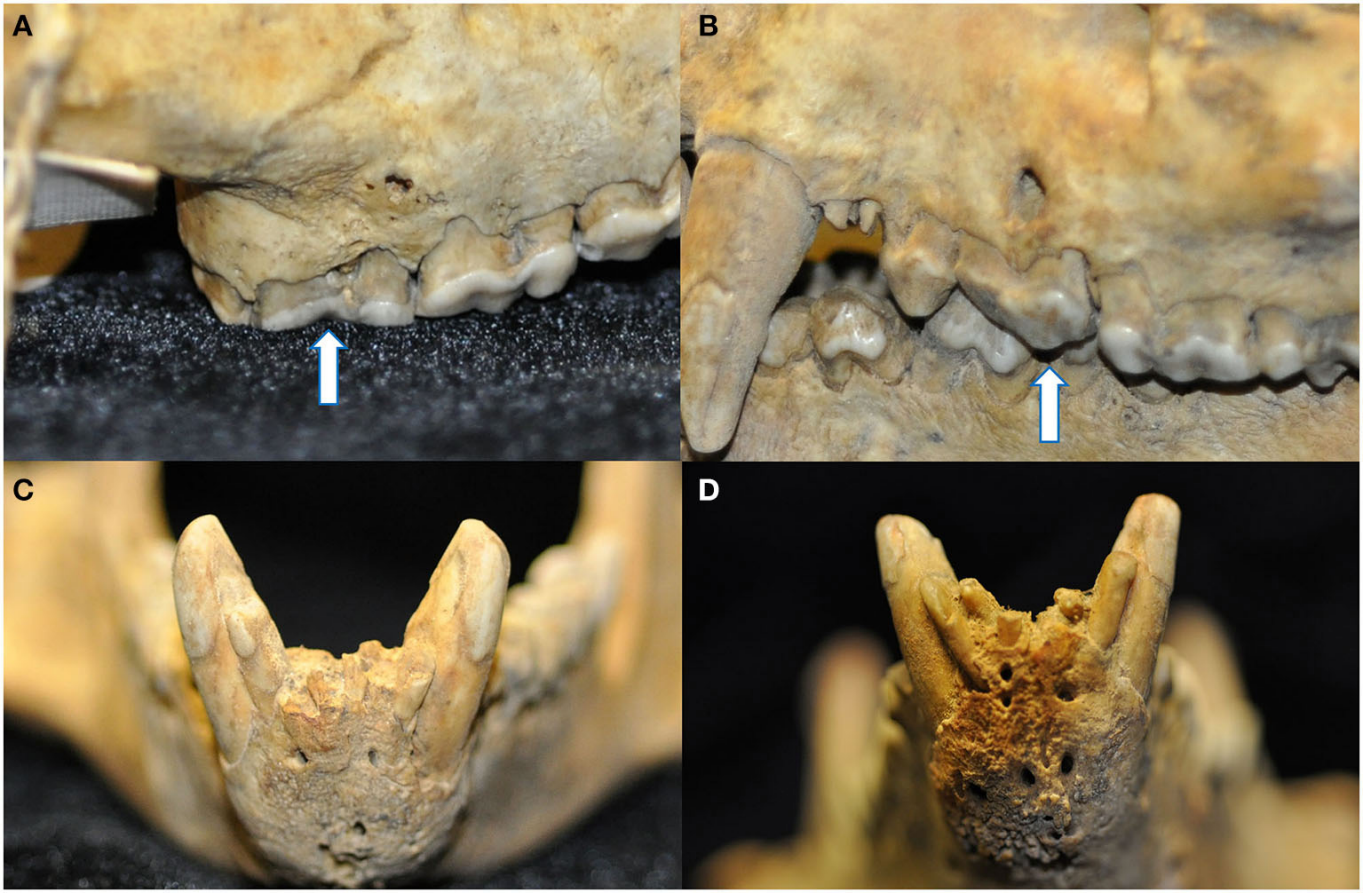
Four instances of pathological changes to the alveolar bone were noted in teeth with radiographic evidence of endodontic disease. Buccal bone recession and periapical fenestration were noted in a discolored right maxillary third molar tooth (arrow) **(A)**, as well as interradicular buccal bone fenestration in a left maxillary third premolar tooth (arrow) **(B)**, and finally mild **(C)** and marked **(D)** abnormal thickening and corrugated irregularity of the alveolar bone of the rostral mandible in association with non-vital mandibular incisor teeth.

Despite the high prevalence of teeth with a loss of crown integrity or fracture line, only 31 teeth (4.5%) demonstrated other radiographic signs of endodontic disease (Figure 8). These included teeth with abrasions (*n* = 10), complicated crown fractures (*n* = 9), root fractures (*n* = 6), linear fractures (*n* = 4), complicated crown root fractures (*n* = 3), and uncomplicated crown fractures (*n* = 2). Of the endodontically compromised teeth with linear fractures, one also had an abrasion, another had an uncomplicated crown fracture, a third had a complicated crown fracture. Mandibular incisor teeth were the most affected (*n* = 10), followed by maxillary incisor teeth (*n* = 5), maxillary canine teeth (*n* = 4), and then one mandibular canine, maxillary premolar, mandibular premolar, and mandibular molar tooth. Only four endodontically diseased teeth had gross alveolar bone changes visible macroscopically (Figure 9). Twenty-seven of the 28 skulls contained fractured teeth without overt radiographic endodontic disease.

**Figure 10:**
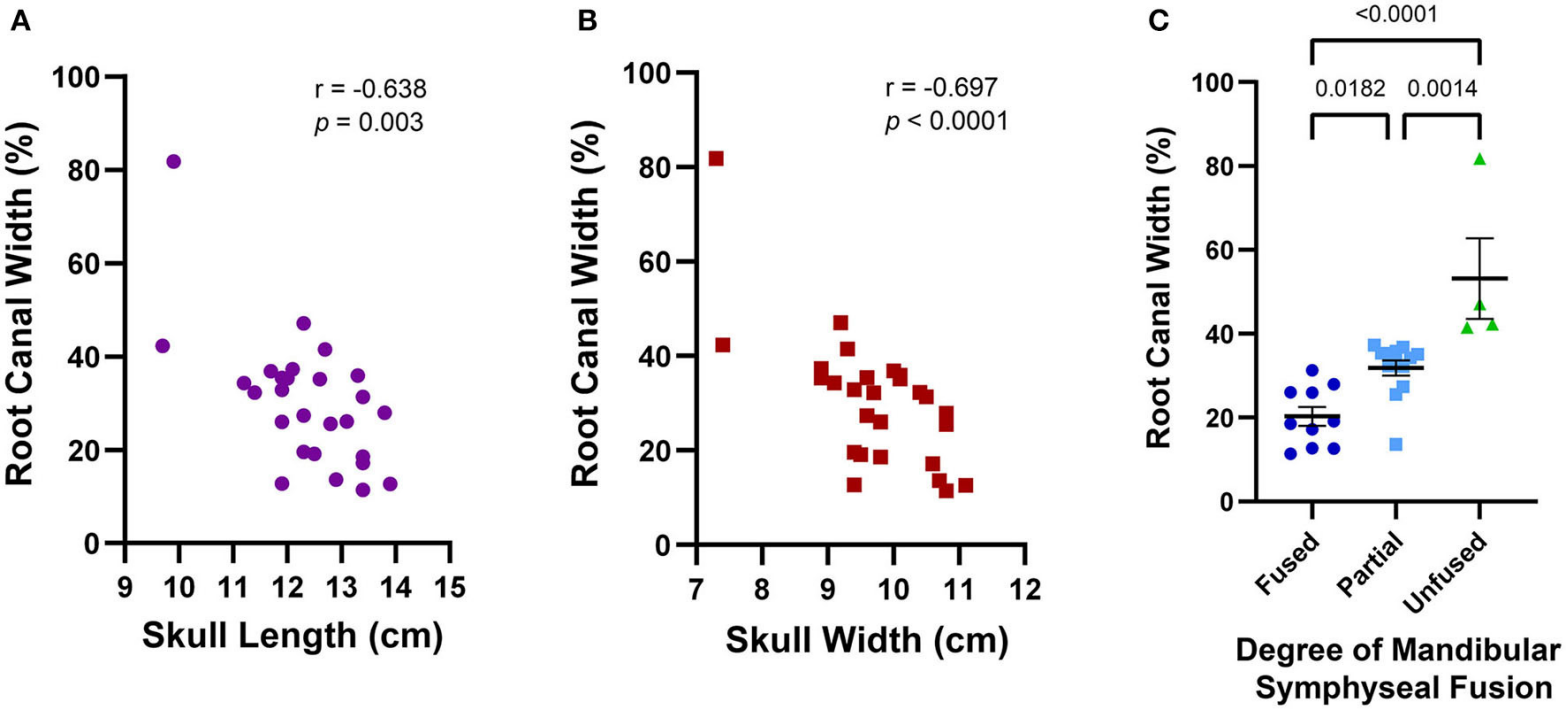
Average maxillary root canal width was found to be associated with skull features that together establish a criteria framework for macroscopic and radiographic age assessment. A wider root canal was significantly associated with a smaller skull length **(A)**, a smaller skull width **(B)**, and a decreasing degree of mandibular symphyseal fusion **(C)**. A wider root canal was also significantly associated with the presence of infraerupted teeth, but since only two of the 28 skulls analyzed contained infraerupted teeth, this data was not separately depicted.

The average maxillary canine root canal width was 30.3% (±14.0%) ranging from 11.4 to 81.8%. Excluding the outlier of 81.8%, the mean root canal width was 28% with a standard deviation of 9.9%. A wider root canal was more likely to be associated with a smaller skull length (*r* = −0.638, *p* = 0.003) and a smaller skull width (*r* = −0.697, *p* < 0.0001) (Figures 10A,B). Skulls with incompletely erupted teeth (*n* = 2) had significantly wider root canals (*p* = 0.0003). Skulls with unfused mandibular symphyses (*n* = 4) had significantly wider root canals than those with partially (*n* = 13) or completely fused (*n* = 10) symphyses, and those with partially fused symphyses had significantly wider root canals than those with completely fused symphyses [*F*_(2,24)_ = 18.06, *p* < 0.0001] (Figure 10C). However, there was no significant association between root canal width and malocclusion (*p* = 0.201), frequency of relative asymmetrical alveolar bone loss (*p* = 0.754), furcation involvement or exposure (*p* = 0.665), or findings consistent with endodontic disease such as relative failure of the root canal to narrow, periapical lucency, or inflammatory root resorption (*p* = 0.251).

**Figure 11:**
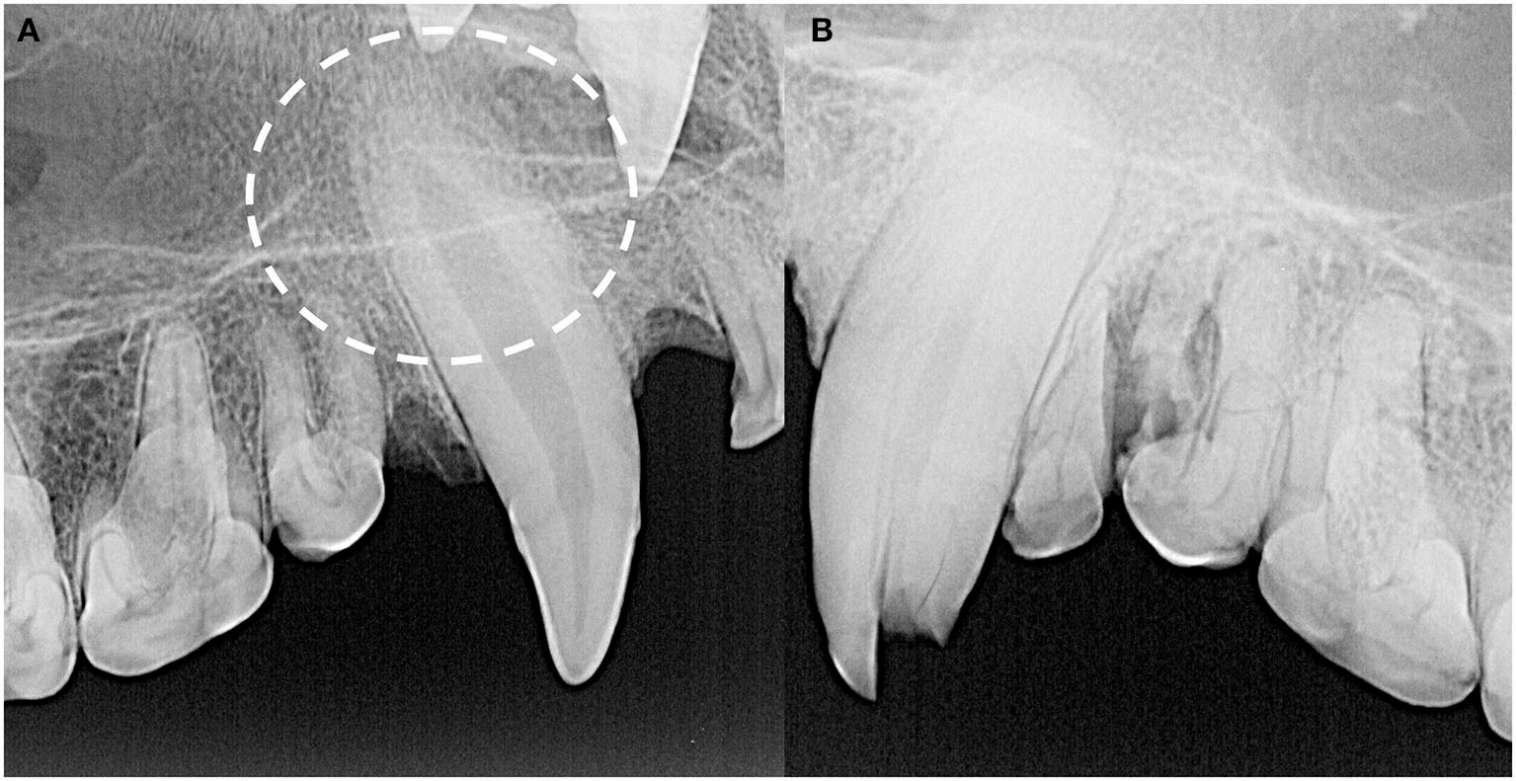
The majority of idiopathic tooth resorption was external replacement resorption affecting predominantly the maxillary and mandibular canine teeth, indicated by the circle **(A)**, but was also noted in premolar and molar teeth. External cervical root surface resorption was also identified affecting a left maxillary third premolar tooth **(B)**.

Finally, tooth resorption of idiopathic origin, as opposed to external inflammatory root resorption from endodontic disease, was identified in 21 teeth (Figure 11) Eighteen of these were classified as external replacement resorption, affecting the maxillary canine teeth (*n* = 7), maxillary third premolar teeth (*n* = 2), maxillary third molar teeth (*n* = 2), mandibular canine teeth (*n* = 6), and mandibular fourth molar teeth (*n* = 3). One case of external cervical root surface resorption of a left maxillary third premolar tooth was also identified.

## Discussion

The 30 Tasmanian devil skulls examined in this study were acquired by the Australian Museum across a period spanning over a 100 years, nearly all of which were preserved to a degree that permitted a thorough macroscopic and radiographic evaluation of skull and dental features. However, a lack of definitive demographic data of these specimens limits the capacity to draw meaningful conclusions regarding precise sex-specific or age-related anatomical and pathological findings. Sexual dimorphism in size in the extant Tasmanian devil is well-documented in the literature, with the male being larger than the female with regards to overall body weight and dimensions (1–5, 8, 9). Greater skull size has been documented in Tasmanian devils up to 30 months of age, as well as in males for the extinct dasyurid *Sarcophilus laniarius* (23, 30). The confirmed female specimens in the present study had measurements at the lower end of the age range in previously documented Tasmanian devil skulls (30). While an initial attempt was made to assess for sexual dimorphism in skull length, skull width, and skull index in the present study, the lack of verified data regarding the sex of the specimens precluded meaningful analysis.

Historical methods for morphometric skull analysis in dasyurids include measurement of condylobasal length and zygomatic arch width, which are analogous to our skull length and width respectively, which is documented to increase with age (23, 30). Other parameters that have not been delineated by age in the literature include length and width of the temporal fossa, width of the postorbital constriction, length of the jaw and dental row, moment arms of the temporalis and masseter muscles, masseteric fossa length, and occipital height (8, 9). The dental eruption pattern of the Tasmanian devil has been described, with the permanent dentition generally erupting in a front-to-rear sequence except for the first maxillary incisors which are usually the last of the incisors to erupt, but considerable variation has been documented between individuals (16, 31). Like other mammals with brachydont dentition, the pulp cavity of the Tasmanian devil narrows with age as layers of dentin are deposited along the lateral walls of the root canal by odontoblasts over the life of the tooth (16, 17). However, to the authors' knowledge there has been no previously described radiographic documentation of narrowing of the root canals with time or progressive mandibular symphyseal fusion in this species.

Root canal width may be considered as a potential means of relative age determination. The coincidence of a wider root canal width with smaller skull morphometrics in this study reinforces the likelihood of a consistent rate of decreasing root canal width reflecting an increase in skull dimensions and corresponding animal age, however future studies with individuals of known age will be required to precisely document this effect. Similarly, patterns of mandibular symphyseal fusion may be used as a means of age estimation in a clinical setting when radiography may be performed in the treatment of clinical disease such as Devil facial tumor disease as this parameter may be unaffected in the face of other compromised maxillofacial anatomy. If validated by a subsequent study involving specimens of confirmed exact ages, these features can provide valuable guidelines for the estimation of patient age.

With regards to normal anatomical skull features, the significance of incongruent sizes of palatine fissures and vacuities is unknown. The thinness of the bone in this location also made it difficult to determine whether some of these differences were due to the result of trauma during postmortem handling and may require histopathologic analysis for definitive assessment. Although found in many vertebrate taxa, the function of the palatine vacuities is not well-defined, apart from anuran amphibians that retract their globes into relatively large palatine vacuities during swallowing (32, 33). Whole head specimen dissection or diagnostic imaging assessment such as computed tomography may be helpful in identification of other anatomical features such as the presence of neural, vascular, or other structures penetrate these palatine openings and thus inform about their function. To the authors' knowledge, this is the first description of the variation in loss of nasal turbinate architecture in Tasmanian devil skulls, as well as deviation of the nasal septum, but due to the delicate nature of these bones and the age of many of these specimens, it is unknown how much of this can be attributed to postmortem loss. Comparison with living or more recent specimens would be valuable to determine the true incidence and clinical significance of such findings.

A variety of novel skull traumas have been described in the present study. However, the lack of callus formation or alveolar bone changes suggestive of osseous disease or remodeling supports the conclusion that these were sustained during postmortem processing, travel, display, or other handling of the skull. This also provides a suspecting cause for the absence of most of the missing teeth and teeth with linear fractures in this study. In the authors' experience, linear dental fractures are a common finding in skull specimens and may be attributed to postmortem changes in the dental hard tissues possibly exacerbated by trauma during handling. However, given the time that had passed between initial specimen inclusion in the museum collection and this study, these skulls were in remarkably good condition which was conducive to more detailed analysis of dentoalveolar findings.

Although suspected fifth molar teeth have been seen in a previous assessment of Tasmanian devil dentition, none of these and no other supernumerary teeth were noted in the present study (21). Among the dental anatomical and positional variations identified in this study, palatal rotation of the mesial aspect of the maxillary premolar teeth and buccal rotation of the mesial aspect of the mandibular premolar teeth was the most common finding, consistent with previous reports (13, 16, 18–21). Because all mandibular premolar teeth in all specimens were rotated buccally relative to the main axis of the mandibular dentition between the canine and fourth molar, this likely represents an anatomical standard for this species. This is in comparison to the maxillary premolar teeth, where it can instead be said that there is a normal variation in the degrees of rotation of these teeth. No overt osseous trauma was seen consequential to these deviations from the main axis of the teeth but use of skulls in this study precludes assessment of potential soft tissue trauma which can be of significant clinical importance. The different crown shape and mesial root thickness of the mandibular fourth molar tooth seen in all specimens is similar in appearance to the crown and root structure of the mandibular molar tooth of cats, which according to a study on molar shape and its relationship to feeding function in carnivores and marsupials reflects their comparable function (34).

To the authors' knowledge, this is the first study to describe normal dental anatomy in the Tasmanian devil using intraoral radiography. Diagnostic imaging is always indicated and often critical for the diagnosis of many anatomical, developmental, periodontal, endodontic, traumatic, oncologic, and other pathologies affecting the dentition, alveolar bone, and bone of the jaws (16, 35). Dental radiographs provide clinically useful information that can inform surgical planning and decrease the risk of intraoperative complications. While no supernumerary or fused roots were identified in the present study, radiographic interpretation of root count and orientation can be more difficult due to the rotation of the premolar teeth, as well root convergence and summation in the maxillary fourth molar teeth. In this case, assessment of the tooth may be done based on macroscopic crown morphology and periodontal probing, but significant subgingival findings such as root convergence and dilacerations that are pertinent to extractions may require more advanced imaging such as medical or cone-beam computed tomography to understand the relevant anatomy more perfectly (16). As root dilacerations, a malformed mandibular canine tooth, and a collection of changes affecting the mandibular second incisor to the first premolar tooth were noted in different specimens, dental radiography should be included in baseline diagnostics when planning treatment of oral disease in Tasmanian devils.

The anatomical abnormality affecting the mandibular second incisor to first premolar molar teeth, involving abnormal tooth arrangement, fusion of the crowns and roots of adjacent teeth, and absence of normal periodontal and endodontic development (Figure 7), may represent a developmental defect or an acquired and possibly neoplastic process. One possible explanation is that this is an odontoma, a type of hamartoma of mixed odontogenic epithelium and ectomesenchyme that includes dental hard tissue formation and in its compound form can contain variably differentiated dental-like structures including calcifying enamel and dentin (36). Histopathological analysis would be needed to confirm this suspicion, as determined by the presence of well-differentiated dentinal tissue including dentin, enamel matrix, odontogenic epithelium representing the enamel organ, and/or cementum (37).

In nearly all teeth in the present study, including the two skulls demonstrating other characteristics of a very young age at the time of death, the margin of the alveolar bone did not reach coronally to the level of the cementoenamel junction. In the domestic dog and cat, this would be classified as pathological alveolar bone loss most commonly secondary to periodontal disease (28). In the absence of soft tissue and the context of clinical findings, we are unable to determine the precise involvement of periodontitis in this phenomenon. While it is known that enamel does not cover the entire crowns of the incisor and canine teeth in this species, it is unknown what degree of alveolar bone presence is considered within normal anatomical variation vs. pathological loss (16). However, it is noted anecdotally that the teeth of dasyurids continue to erupt to some degree throughout the lifetime of the animal, and the cementoenamel junction may move progressively coronally relative to the alveolar bone as a part of normal tooth eruption and aging (23). As such, the nine maxillary fourth molar teeth which had alveolar bone reaching the cementoenamel junction may indicate these specimens were juveniles to young adults, as opposed to reflecting a non-pathologic anatomical standard. The mutually exclusive nature of assessing relative asymmetrical alveolar bone loss means that this should not be used as a sole method of identifying potential pathological alveolar bone loss. This analysis also assumes the plaque-retentive consequences of alveolar bone recession from the furcation in periodontal disease in a comparable manner to that of the domestic dog and cat, but once again, the clinical consequences of this finding are unknown. Further study with precise measurement of suprabony cemental exposure coupled with soft tissue findings would be needed to quantify what degree of this relative alveolar bone recession is considered clinically normal.

In this study, most teeth demonstrating a loss of crown integrity had abrasive wear. This abrasion is likely attributable to the diet of the Tasmanian devil, specifically the tendency of this species to consume whole prey including mastication and ingestion of the bones (11, 12). This may also account for the relative rarity of enamel fractures documented in this collection, as enamel fracture edges may quickly become smoothened and be visually difficult to differentiate from abrasions. The relatively increased frequency of incisor teeth demonstrating radiographic signs of endodontic disease may be due to an increased likelihood of trauma during the process of capturing and killing prey items or may simply be a mechanical consequence of having a long tooth height to diameter ratio.

In conclusion, the findings of this study can help develop a foundation for understanding the skull and dental anatomy and disease processes of Tasmanian devils. This knowledge can guide the management of oral health in live animals, including captive specimens that may play an important role in a recovery plan for Tasmanian devils. The utility of dental radiographs in detecting possible periodontal and endodontic disease can be important in the captive management and veterinary care of Tasmanian devils who may not demonstrate overt outward signs of pain. Even so, it is important to bear in mind that these skulls are not reflective of a given population due to the staggering of collection times, uncertainty of location of origin for some specimens, and also the possibility that animals with these pathologies may be overrepresented due to decreased fitness compared to local age-matched conspecifics leading to a potential restriction in access to nutrition, decreased ability to defend themselves, and potentially decreased breeding opportunities. The next logical step would be to validate these findings on a larger collection of skulls with more complete patient demographic data, and to ultimately start collecting these values on live individuals under general anesthesia with known clinical histories, with the opportunity to assess follow-up of medical treatments.

## Data Availability Statement

The raw data supporting the conclusions of this article will be made available by the authors, without undue reservation.

## Author Contributions

SL prepared the manuscript. NF and SP contributed equally to its conceptual development and editorial process. LV facilitated and supervised data collection and reviewed the manuscript. All authors contributed to the article and approved the submitted version.

## Conflict of Interest

The authors declare that the research was conducted in the absence of any commercial or financial relationships that could be construed as a potential conflict of interest.
